# Shoulder Osteonecrosis: Pathogenesis, Causes, Clinical Evaluation, Imaging, and Classification

**DOI:** 10.1111/os.12788

**Published:** 2020-10-04

**Authors:** Philippe Hernigou, Jacques Hernigou, Marius Scarlat

**Affiliations:** ^1^ Henri Mondor Hospital University Paris Créteil France; ^2^ EpiCURA‐ Baudour Hornu Hospital Mons Belgium; ^3^ Clinique St. Michel Groupe ELSAN Toulon France

**Keywords:** Corticosteroids shoulder osteonecrosis, Dysbarism shoulder osteonecrosis, Humeral head osteonecrosis, Posttraumatic humeral head osteonecrosis, Sickle cell disease shoulder osteonecrosis

## Abstract

The humeral head is the second most common site for nontraumatic osteonecrosis after the femoral head, yet it has attracted relatively little attention. Osteonecrosis is associated with many conditions, such as traumatism, corticosteroid use, sickle cell disease, alcoholism, dysbarism (or caisson disease), and Gaucher's disease. The diagnosis is clinical and radiographic with MRI, with radiographs being the basis for staging. Many theories have been proposed to decipher the mechanism behind the development of osteonecrosis, but none have been proven. Because osteonecrosis may affect patients with a variety of risk factors, it is important that caregivers have a heightened index of suspicion. Early detection may affect prognosis because prognosis is dependent on the stage and location of the disease. In particular, the disease should be suspected in patients with a history of fractures, steroid usage, or sickle cell disease, and in divers. This report reviews osteonecrosis of the humeral head, with an emphasis on causes, clinical evaluation, imaging, and classification.

## Graphical abstract

The humeral head is the second site for osteonecrosis; it is associated with traumatism, corticosteroid use, sickle‐cell disease, alcoholism, dysbarism (or caisson disease), Gaucher's disease, and other conditions.

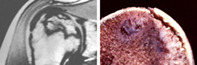

## Introduction

**Fig. 1:**
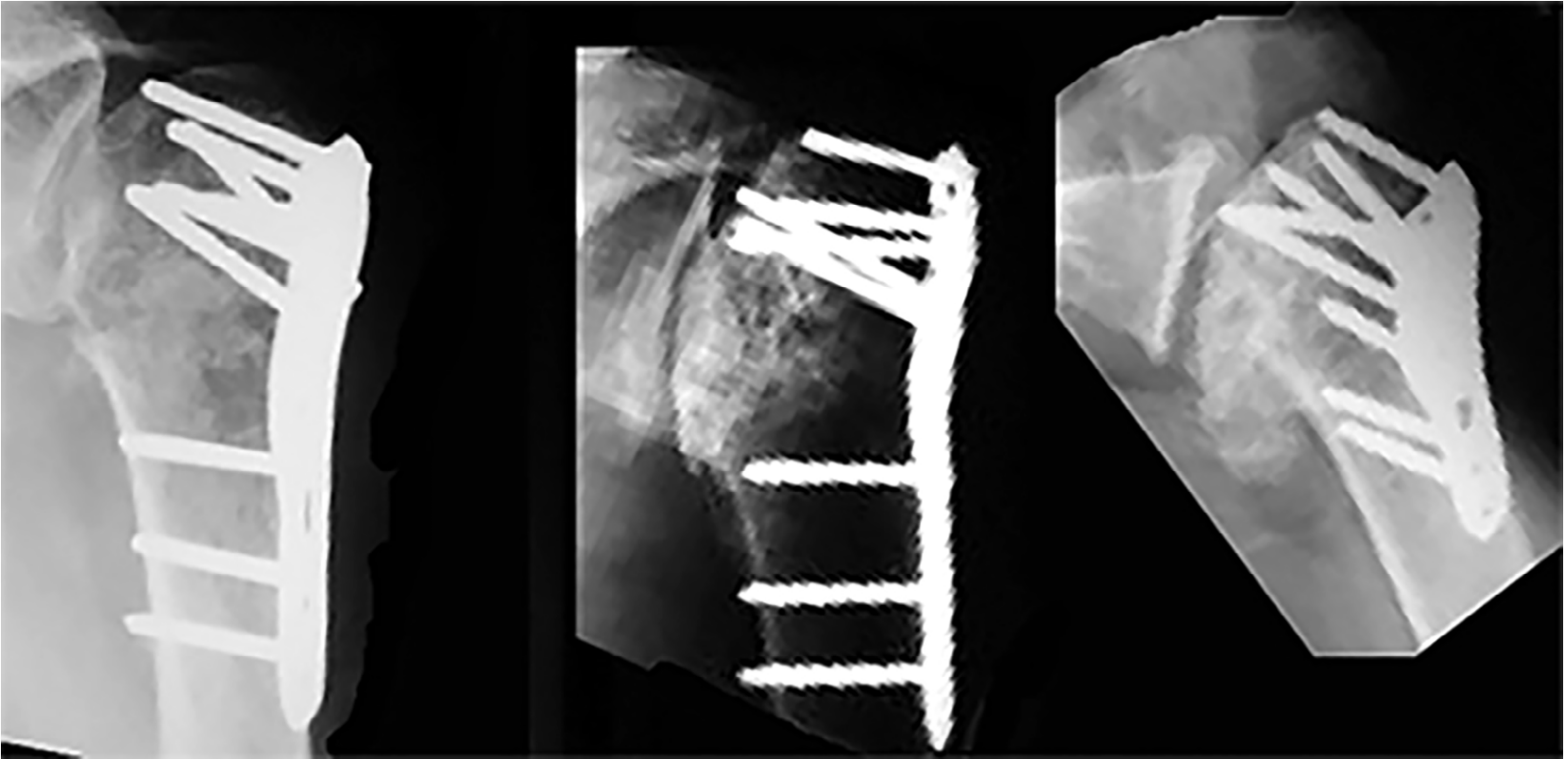
Posttraumatic radiograph of humeral head and evolution to osteonecrosis.

After the femoral head, the humeral head is the most frequent location for nontraumatic osteonecrosis^1^. This pathology is underestimated, and a proper clinical evaluation must be presented as knowledge for the orthopaedic surgeon. In addition to post‐traumatic avascular osteonecrosis (AVN)^2^, causes of humeral head necrosis (Fig. 1) are sickle cell disease (SCD)^3^, the first genetic disease in the world, and corticosteroid therapy^4^; as a result, a large population of patients are at high risk for shoulder osteonecrosis. Isolated osteonecrosis of the humeral head in the absence of involvement of the hip is rare; in fact, it is frequent in patients with multifocal osteonecrosis^5^, ^6^.

## History of Humeral Head Osteonecrosis

### *Shoulder Osteonecrosis: A 250 Million Year History*

Mesozoic diving marine reptiles have been characterized on the basis of avascular necrosis caused by decompression. The ichthyopterygians reptiles appeared around 250 Ma; they were marine reptiles and became extinct 90 Ma^7^. The humeral head revealed avascular bone necrosis in 16% of these reptiles. Bone death was caused by intravascular bubble formation during decompression. After deep diving and during decompression, nitrogen changes from dissolved phase to free phase and gas bubbles may be formed in many tissues. As nitrogen is more soluble in fat^7^, it will dissolve in the fatty marrow of the long bones. Normally, bubbles are formed during decompression in the venous circuit, and the lung is effective as a filter. This is true when the systemic and the pulmonary circuit are separated, as for mammals and whales. However, an opening in the heart with the possibility of right to left shunting in those reptiles allowed venous nitrogen‐oversaturated blood to flow into the systemic circulation, compromising the vascularization of bone.

### *Better Knowledge in the 20th and the 21st Century*

Dysbaric shoulder osteonecroses were reported as early as 1911 by Bornstein and Plate^8^, followed later by Bassoe in 1913^9^, who presented radiological confirmation of aseptic necrosis of the shoulder in compressed air workers. The humeral head has aroused rather limited research interest as compared with hip osteonecrosis. In 1960, Heimann and Freiberger^10^ provided initial descriptions of shoulder osteonecrosis; in 1968, Cruess *et al*.^11^ presented a classification of shoulder osteonecrosis. More recent studies have reported on patients with humeral head osteonecrosis.

## Etiology and Risk Factors

There are many causes of humeral head osteonecrosis (Table 1) described in the literature. We will focus on the most frequent.

**Table 1:** Causes of humeral head osteonecrosis

Trauma
Fractures
Fracture–dislocations
Non‐traumatic conditions
Sickle‐cell anemia
Metabolic/endocrinologic
Gaucher disease
Rheumatologic disorders
Systemic lupus erythematosus
Extrinsic factors
Dysbaric conditions (Caisson disease)
Alcohol consumption
Iatrogenic
Corticosteroids
Organ transplantation

### *Trauma*

**Fig. 2:**
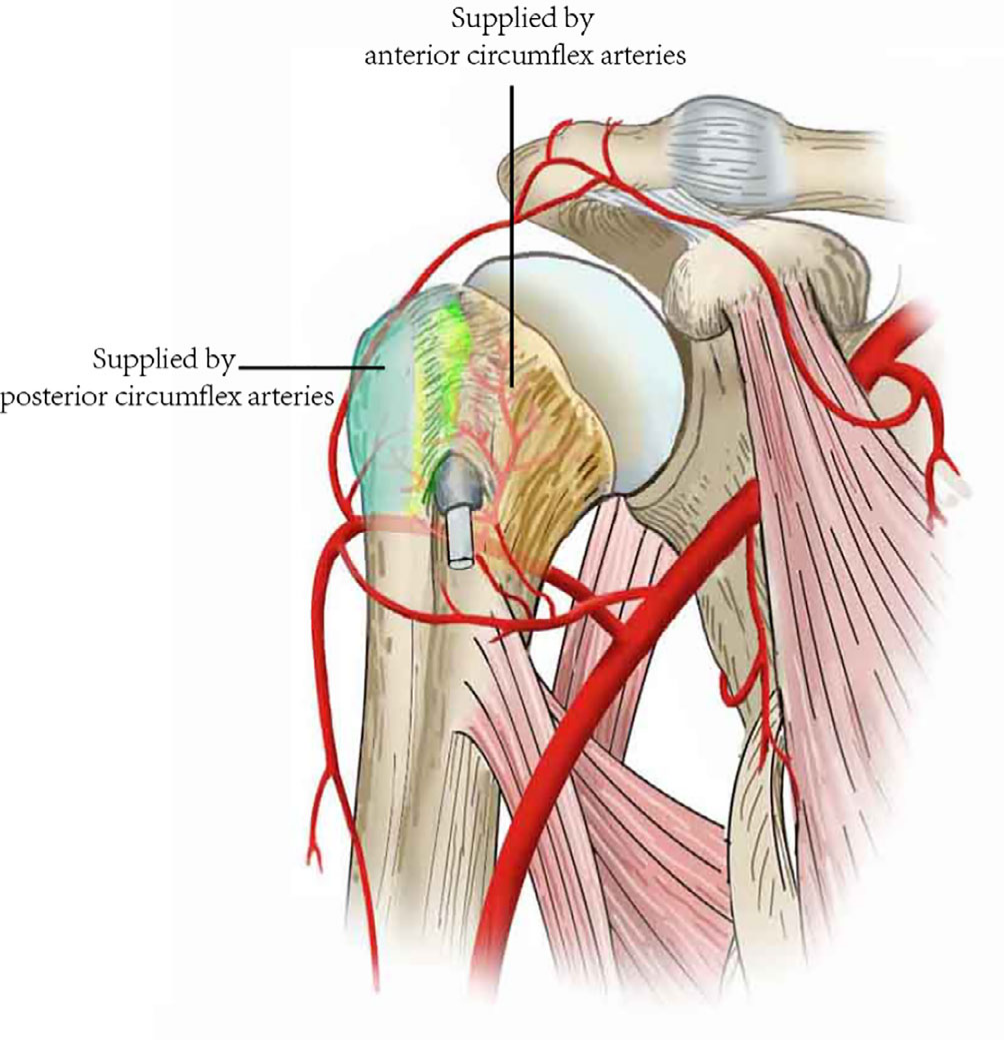
Vascularization of the humeral head.

Trauma leads to humeral head osteonecrosis by disrupting the vascular supply. Humeral head osteonecrosis is frequently identified in patients who have undergone internal fixation (Fig. 1). This occurs when the blood supply to the humeral head is disrupted by a fracture. Vascular anatomy allows us to understand the mechanism of necrosis. The ascending branch of the anterior humeral circumflex artery is thought to be predominant for vascularization ^12^. This finding was supported by Gerber *et al*. in 1990^13^. They also showed that, through anastomoses of these two arteries (Fig. 2), the anterior circumflex and posterior circumflex arteries supply the humeral head with other arteries. The anterior circumflex artery reaches the surgical neck of the humerus at the inferior border of the subscapularis after passing beneath the coracobrachialis and the short head of the biceps. Despite the posterior circumflex artery having a diameter three times larger than that of the anterior circumflex artery, it supplies only the greater tuberosity and the posteroinferior part of the humeral head.

**Fig. 3:**
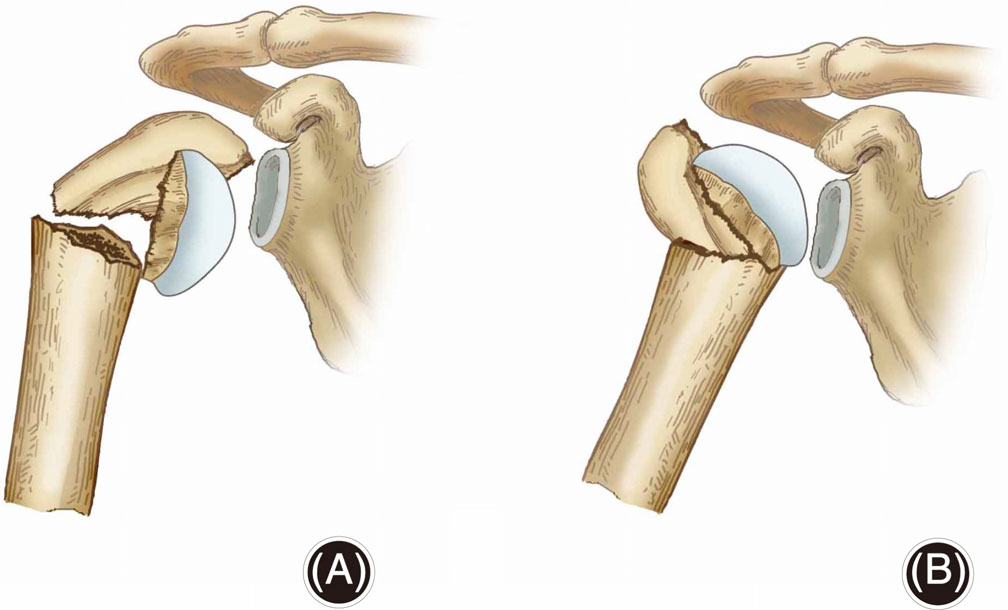
(A) No hinge with high risk of osteonecrosis; (B) hinge (arrow) and low risk of osteonecrosis.

Although it may be suspected that a history of dislocation could lead to osteonecrosis *via* capsular injury and vascular shearing, this has not been proven. Solberg *et al*.^14^ find osteonecrosis to be the most common complication of fractures (16% affected). There is a strong correlation between osteonecrosis and the length of the initial metaphyseal contact point or hinge (Fig. 3) attached to the articular fragment, as demonstrated by Hertel *et al*.^15^. Robinson *et al*.^16^ investigated patients who had sustained proximal humeral fracture dislocations that were treated with osteosynthesis. Robinson proposed as classification for risk of osteonecrosis after fracture the following criteria. Type I injuries: fracture capsular attachment, 2 cm in length, with cancellous bone arterial bleeding. Type II injuries: minimal capsular attachment, <2 cm in length, with no bleeding present. Using this classification, osteonecrosis with type II injuries was more frequent. Robinson *et al*.^16^ also included bone bleeding as a predicting factors; however, some humeral heads with signs of perfusion went on to develop osteonecrosis.

Finally, the age at which the injury occurs may also be an important risk factor, but this is not proven clinically. Patients sustaining three‐part and four‐part fractures have been investigated for humeral head viability through tetracycline labeling^17^, with results suggesting that younger patients are more resistant to ischaemia.

Patel estimated the number of posttraumatic osteonecrosis after fracture ^2^. The risk of osteonecrosis ranges from 0% to 25%^18^, ^19^ for three‐part fractures and 0%–77% for four‐part fractures, with an increased rate with longer follow‐up^20^.

### *Corticosteroids*

Corticosteroids are a common cause of atraumatic humeral head necrosis, but the exact mechanism is unknown. Hernigou^4^ evaluated the natural history and the rate of disease progression with a long‐term follow up. The outcomes of 215 shoulders (125 adult patients) with osteonecrosis related to corticosteroids were evaluated at early stages before collapse with radiographs and MRI. With an average 15 years of follow up (range 10 to 20), there was pain in 65% of asymptomatic shoulders and collapse had occurred in 50% of shoulders. In symptomatic shoulders, collapse has developed in most at the final follow up. The time between the diagnosis and collapse was an average lof 6 years. At the most recent follow up (average 15 years), 50% of shoulders had required surgical treatment. Stage, occurrence of pain, and continuation of peak doses of corticosteroids were risk factors for progression of osteonecrosis.

The role of steroid dose remains controversial. Suspected as early as 1957^21^, whether this is a dose response, threshold, peak‐dose, or idiosyncratic phenomenon is unclear. The high dose of steroids during the first several weeks seems to be more important than the total cumulative dose. In addition, patients may have a predisposition toward osteonecrosis, with a possible genetic susceptibility for the disease.

**Fig. 4:**
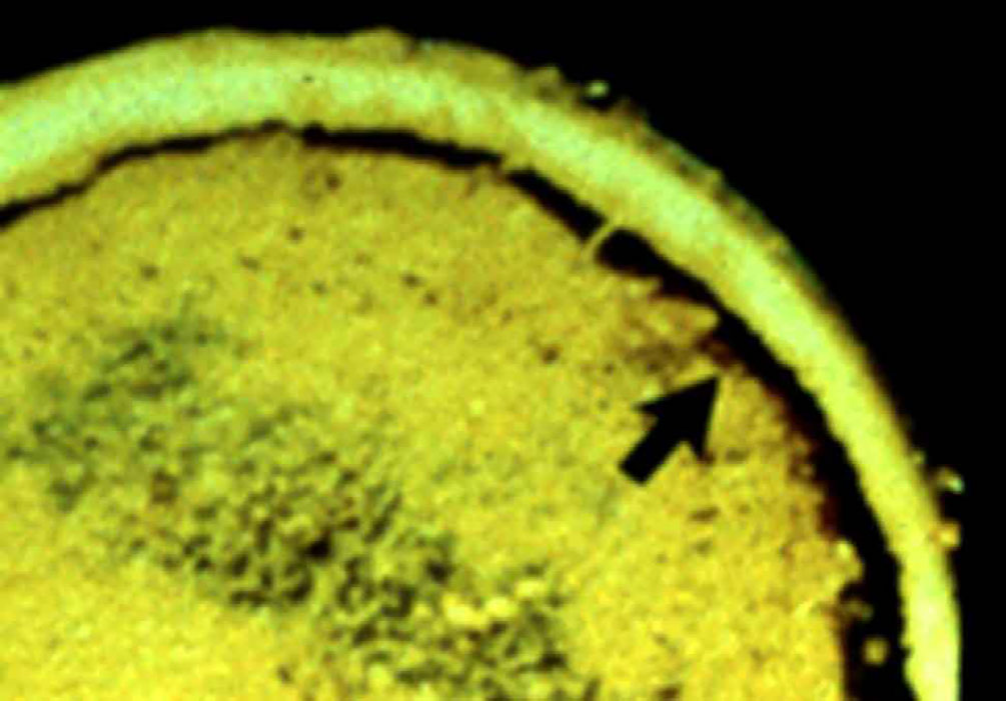
Osteonecrosis related to corticosteroids: accumulation of fat; subchondral fracture indicated by the arrow.

The most commonly accepted theory surrounding corticosteroids and shoulder osteonecrosis involves fat accumulation in marrow (Fig. 4), leading to increased intraosseous hypertension and decreased blood flow. This concept has been extrapolated ^22^ into a multiple‐hit theory in which corticosteroids alter bone homeostasis, injure bone cells, impair blood flow, and suppress bone cell precursors in susceptible patients. Corticosteroids inhibit angiogenesis and promote a hypercoagulable state, which could contribute to the formation of intravascular thrombosis.

### *Sickle Cell Disease*

**Fig. 5:**
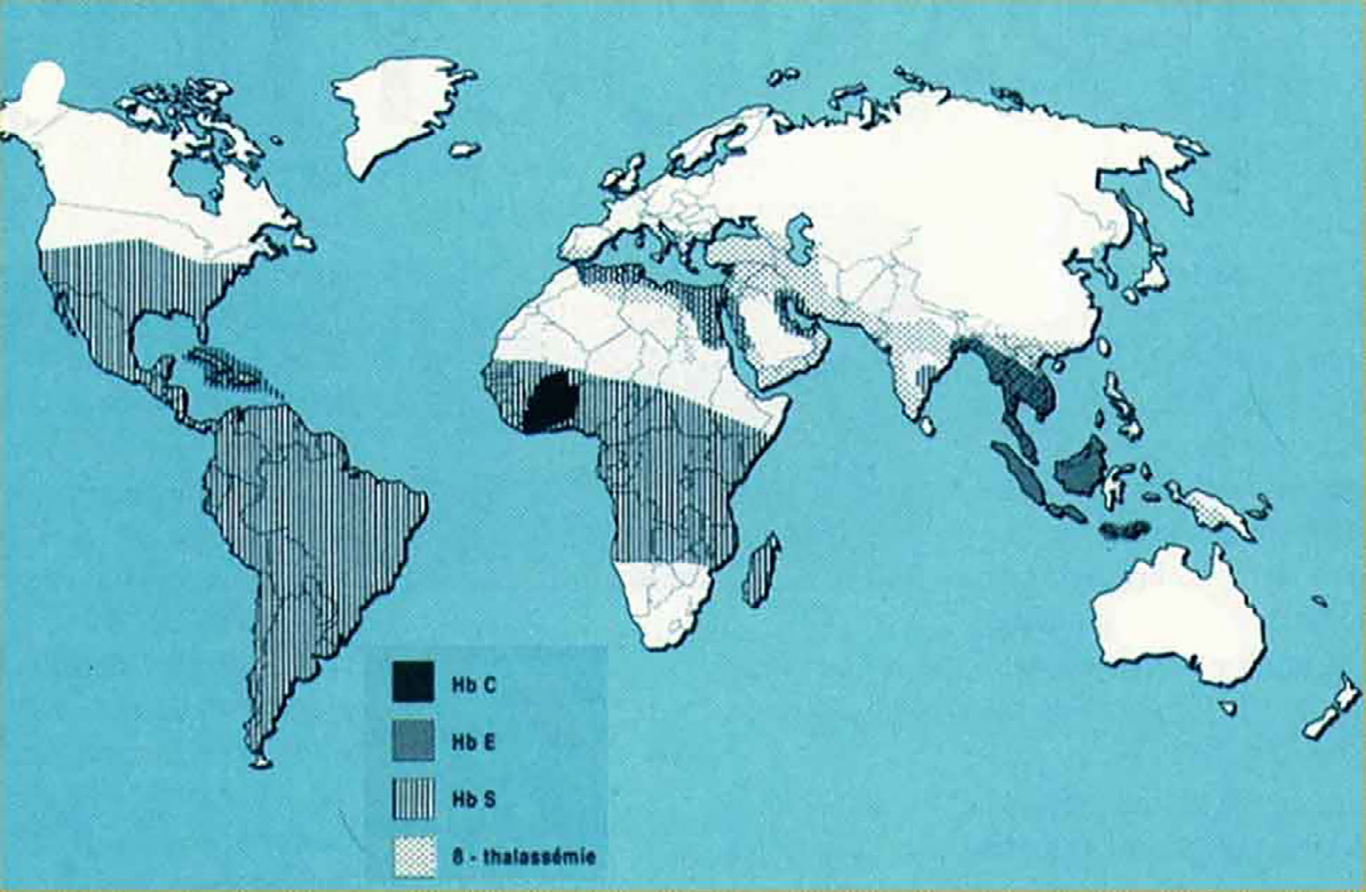
Repartition of sickle cell disease in the world.

**Fig. 6:**
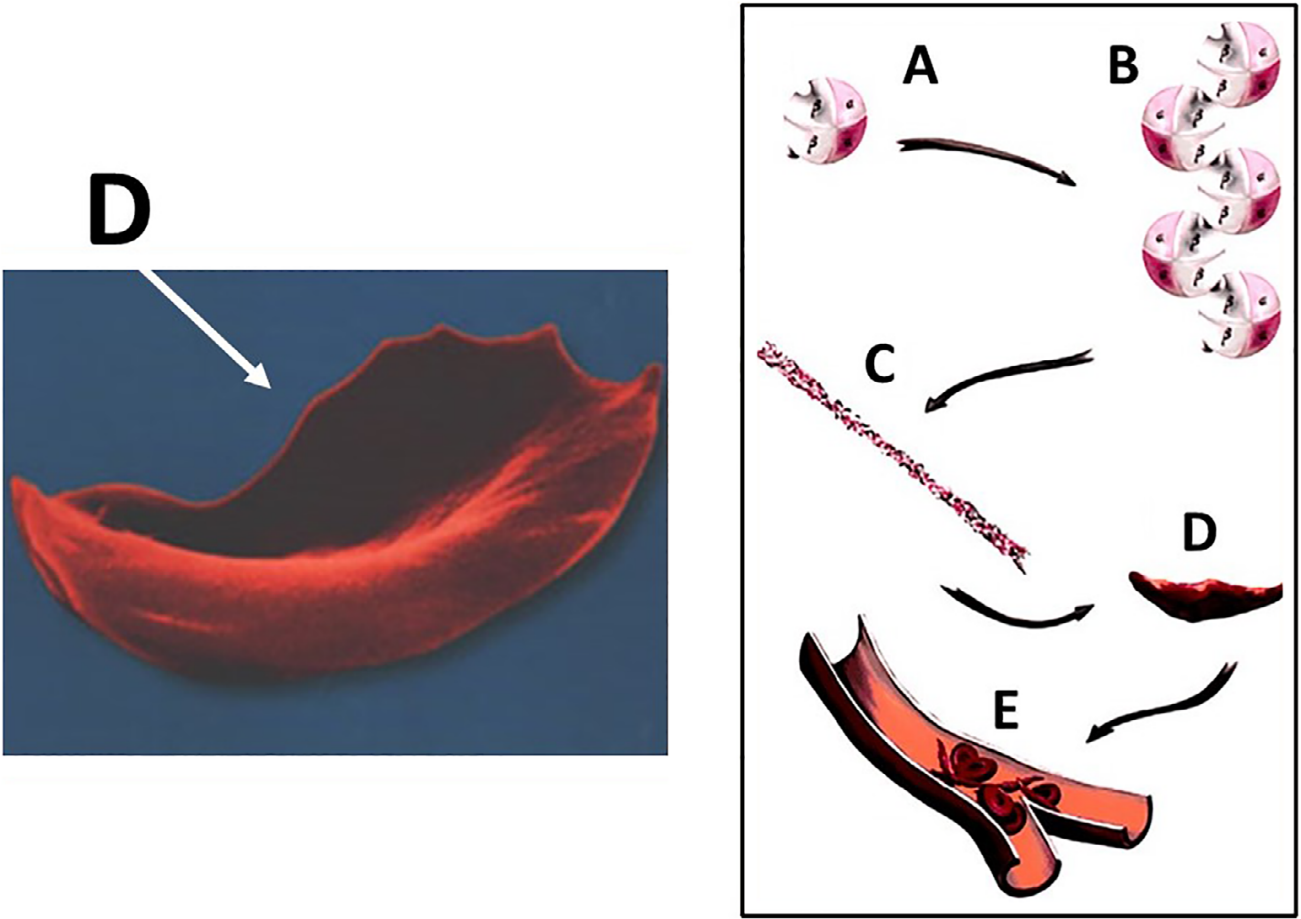
Mechanism of osteonecrosis in sickle cell disease; the polymerization (A and B) of hemoglobin S (in a deoxygenated state) producing cells that are crescent‐shaped or sickle‐shaped (C and D) with decreased deformability; the decreased deformability results in greater risk of clotting (E) in small vessels.

Sickle cell disease, an autosomal recessive disorder, is also called sickle cell anemia (SCA) due to the hemolytic anemia characterized by abnormally shaped (sickled) red blood cells (RBC), which are removed from the circulation and destroyed at increased rates, leading to anemia. Of greater clinical importance, the sickled RBC cause vascular occlusion, which leads to tissue ischemia and infarction. The patients who are homozygous for the sickle cell gene are named “hemoglobin SS” or Hb SS patients. There have been at least four mutational events occurring independently, with three in Africa and a fourth in Saudi Arabia or central India. These events occurred from 3000 to 6000 generations ago, approximately between 70,000 and 150,000 years ago. The distribution of Hb SS in the world (Fig. 5) is indicative of two factors: selection for carriers with survival advantages in malaria‐endemic regions and subsequent migrations. The first descriptions of humeral head osteonecrosis were published by Chung^23^ and Millner^24^. Patients who are homozygous for the sickle cell gene have a high risk of bone osteonecrosis due to microvascular occlusion (Fig. 6) in relation to the disturbance in the erythrocyte architecture.

Although precise data are not available, a recent estimation suggests that SCD affects 0.74% of births in sub‐Saharan Africa. By comparison, only 0.15% of the black population of the United States and of Europe is affected by SCD. SCD is also an important etiology of hip osteonecrosis in the Indian subcontinent, in South America, in the Persian Gulf, in the Mediterranean countries and those from the Caribbean, and in Central America. It is estimated that each year over 300,000 babies (every decade 3 million) with severe forms of these diseases are born worldwide, with the majority in low‐income and middle‐income countries. Approximately 5% of the world's population are healthy carriers of a gene for SCD or thalassaemia. In high‐income countries, the survival of people with SCD has increased steadily, often to adulthood. In contrast, infant mortality related to SCD in Africa remains between 50% and 90%, with less than half of affected children reaching their fifth birthday. An index of the high mortality rate throughout childhood is the observation that the prevalence of Hb‐SS in adults is 10 times lower than the incidence of births (0.2%–0.3% against 2% to 3%). Nearly 90% of the global population with SCD live in three countries: India, Nigeria, and the Democratic Republic of Congo, where 2% of the population is affected by the disease and the prevalence rate of carriers (trait sickle cell) reaches 10% to 30%. Nigeria alone would have at least 150,000 newborns a year. According to these data and to some extrapolations, SCD affects several million adults worldwide. Specific orthopaedic manifestations of SCD and its sequelae include osteonecrosis, infection, and bone marrow hyperplasia. Osteonecrosis of the humeral head (shoulder) has been reported in up to 50% of patients based on the type of hemoglobinopathy. With this frequency (50%) and the presence of bilateral osteonecrosis most often in this population, the number of shoulder osteonecrosis to SCD is probably several milli on in the world, and it is probably the most frequent cause of shoulder osteonecrosis.

Hernigou^25^ and Poignard^26^ reported the natural evolution of 82 adult patients with SCD and osteonecrosis of the humeral head. A total of 104 cases of shoulder osteonecrosis were identified with MRI. Shoulder osteonecroses were graded with MRI and radiographs. Partial or total repair with a decrease in the size of the osteonecrosis or stage regression was never observed on MRI. At average follow up 20 years (range 15 to 24 years), collapse had occurred in 86% of shoulders. The mean interval between onset of pain and collapse was 6 years. The principal risks for shoulder osteonecrosis in adults with SCD were the presence of hip osteonecrosis, and the genotypes S Beta or SC.

### *Alcohol*

The connection between alcohol use and shoulder osteonecrosis has been known for almost a century, and, as with corticosteroids, the exact mechanism is unknown. Animal studies of bone marrow treated with alcohol have shown increased pressure and adipogenesis and decreased hematopoiesis, which could lead to osteocyte injury and shoulder osteonecrosis ^27^. There seems to be a dose response relationship; however, there is no established threshold beyond which patients are proved to be more at risk^28^. Given the relatively low prevalence of shoulder osteonecrosis among all alcohol users, there is likely a multifactorial relationship between shoulder, genetic susceptibility, environmental factors, and medical comorbidities.

### *Dysbaric Osteonecrosis*

**Fig. 7:**
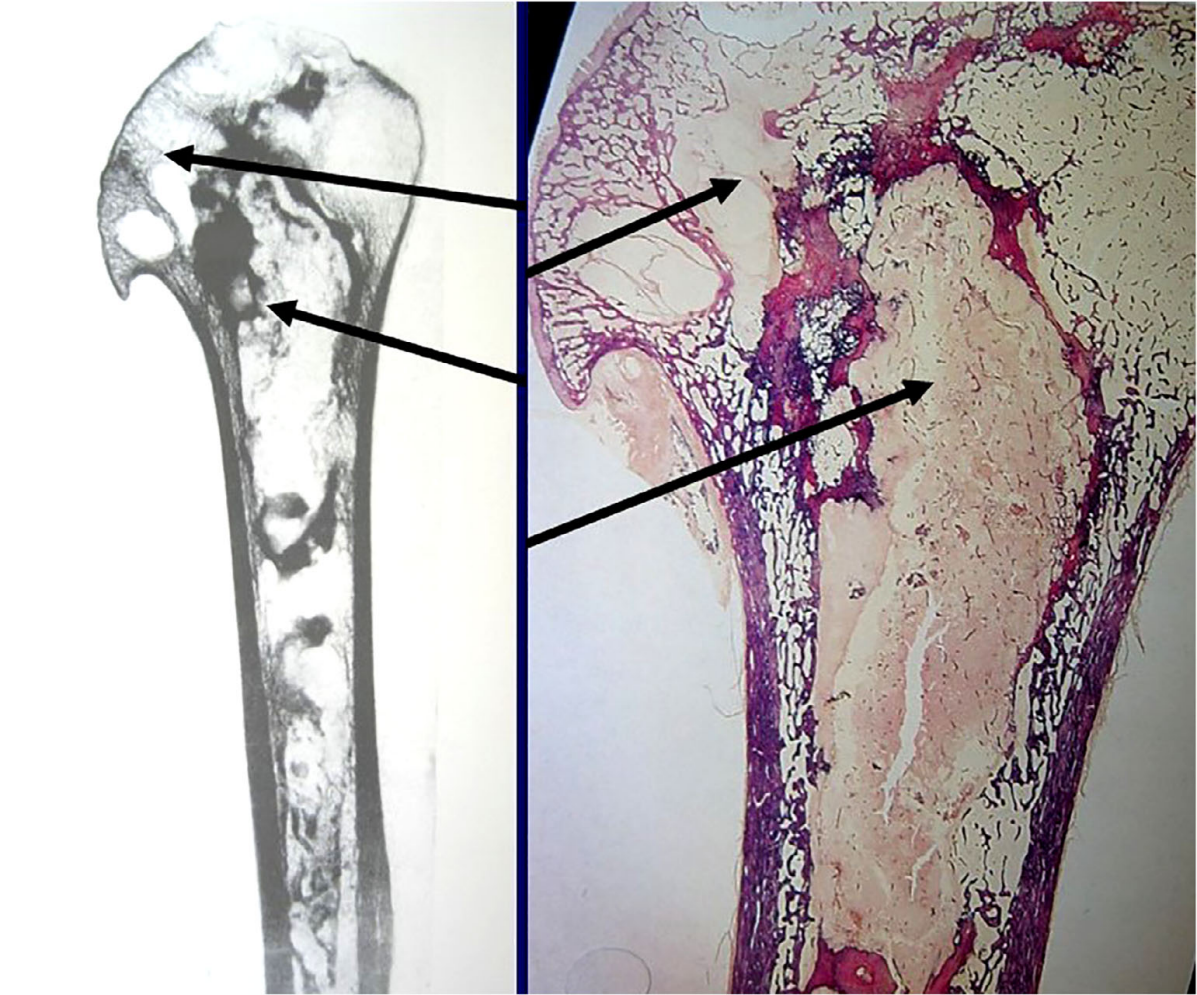
Shoulder osteonecrosis in a diver with a fatal decompression accident of: post mortem images (radiograph and anatomy) of osteonecrosis (arrows) in the proximal humeral head.

The risk of decompression illness^29^ is directly related to the depth of the dive, the amount of time under pressure, and the rate of ascent. Dive tables, such as the US Navy Dive Tables, provide general guidelines as to what depths and dive times are less risky for the development of decompression sickness. In humans, decompression illness is seen among scuba divers and compressed air workers, where the uptake of nitrogen in the blood and tissues is continuously taking place ^30^, ^31^. If appropriate decompression procedures have not been followed, independently of the risk of death, these divers and workers (Fig. 7) will be at risk of avascular bone necrosis ^32^. Although dysbaric osteonecrosis is well known by paleontologists^7^, it is now less well known by orthopaedists because divers use dive tables. However, it is important to understand (and usually rheumatologists and orthopaedic surgeons do not know) that even divers who follow decompression schedules and tables may still experience decompression illness in cases of foramen ovale. The human heart has four chambers, and the systemic and pulmonary circuit is normally fully separated. In the fetus, the left and the right atrium communicate with one another through the foramen ovale. This opening closes during the first weeks of life. In the fetus, the lungs are not functioning and the fetal blood is oxygenated in the placenta. In the atrium, 60% of the blood is shunted from the rightd to the left side (R–L shunt). In some individuals^32^, this intracardiac opening is persistent to some degree in adult life as a patent foramen ovale (PFO). Small‐ and medium‐sized openings are normally symptomless and may be present in as many as 30% of normal subjects. In humans, the risk of decompression illness is five times higher in individuals with patent foramen ovale; this condition allows blood shunting from the venous circuit to the systemic circuit. There is convincing evidence of a connection between PFO and decompression illness in divers; Torti *et al*.^33^ found that PFO was correlated with a five times increased risk of major decompression illness events and the risk augments with the PFO size.

Situations that elevate right atrial pressure relative to left atrial pressure would increase the tendency for any right to left shunting, such as breath holding, coughing, and the Valsalva maneuver (all common during scuba diving). The Valsalva technique, used to equilibrate the pressure in the middle ear, increases pressure in the right atrium and thereby increases the right–left shunt if PFO is present.

### *Miscellaneous*

Many other factors have been associated with shoulder necrosis. Gaucher disease is a disorder where an enzyme deficiency causes accumulation of glucocerebroside in macrophages. The result is infiltration of the bone marrow with vascular compromise. A study of patients with Gaucher disease and shoulder necrosisfound that it was more prevalent in patients who had a prior splenectomy ^34^.

## Clinical Evaluation

Insidious pain with movement is frequent in nontraumatic necrosis of the humeral head. Pain at night is not frequent; however, in one study, 70% of patients had difficulty sleeping^35^. Symptoms may prevent physical work in up to 80% of patients. A “click accompanying certain movements” results from joint incongruity, cartilage flap, or a large loose body. Patients with osteonecrosis of the humeral head are usually younger than patients with osteoarthritis.

Physical examination may reveal tenderness, but motion is often preserved until the late stages. The discomfort is greater with the arm abducted or elevated 90°, corresponding to maximum glenohumeral loading.

Depending on the history, useful tests may include blood count, erythrocyte sedimentation rate, and C‐reactive protein to help rule out infection. Specific serology for rheumatoid arthritis is useful to rule out inflammatory conditions. SCD is confirmed by analysis of hemoglobin. Gaucher's disease is characterized by elevated serum acid phosphatase, but the diagnosis should be confirmed by enzymatic and mutational analysis. In many cases, the history, physical examination, and laboratory tests serve only to raise the suspicion of osteonecrosis.

## Imaging

If history and physical examination findings are suspicious for shoulder osteonecrosis, plain radiography is the next step in diagnosis. Although very early shoulder osteonecrosis may be undetectable on plain radiography, early shoulder osteonecrosis shows cystic and/or sclerotic changes in the humeral head. The term “crescent sign” describes an area of subchondral lucency in the humeral head that indicates subchondral fracture due to bone necrosis and subsequent attempts at repair. shoulder osteonecrosis at a later stage shows humeral head flattening, collapse, and degenerative changes.

**Fig. 8:**
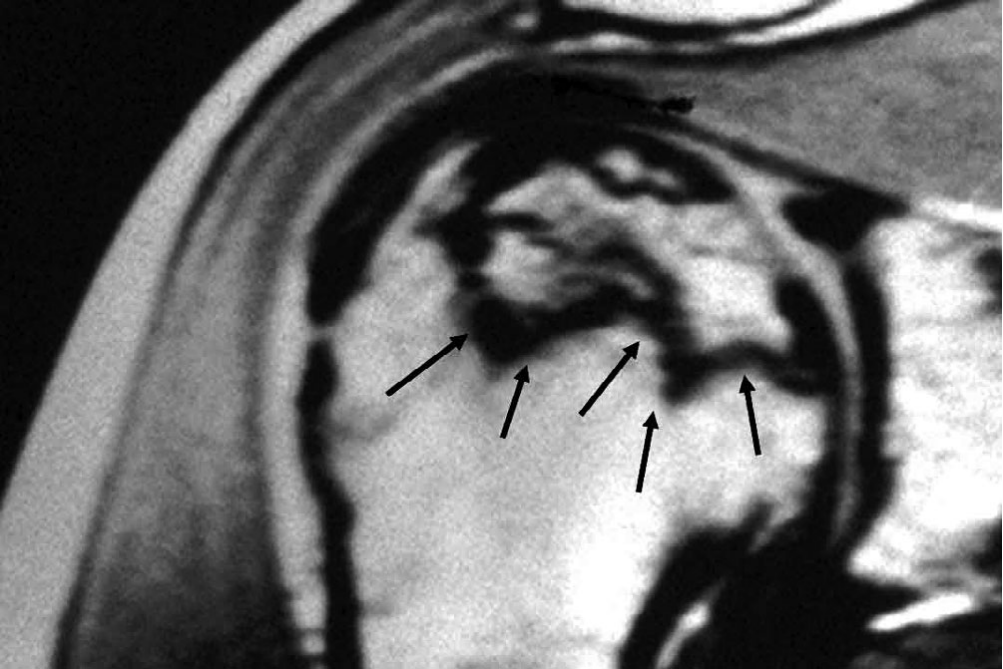
MRI appearance of early shoulder osteonecrosis: band‐like hypo‐intense (arrow) zones on T1‐weighted images demarcating the osteonecrosis.

**Fig. 9:**
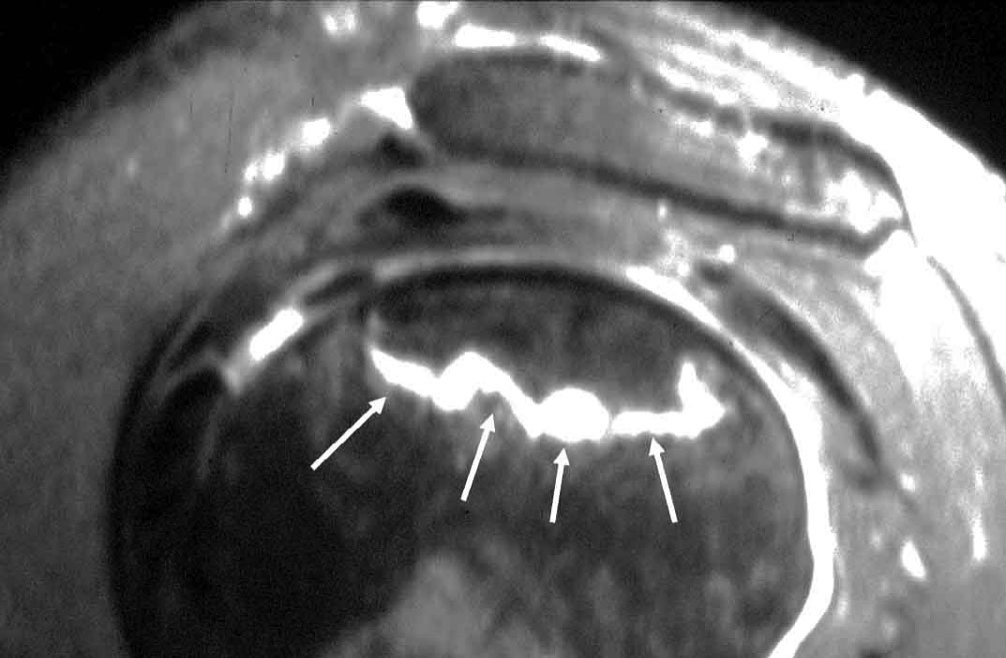
Area of high‐intensity signal on T2‐weighted images of osteonecrosis (arrow) on MRI.

MRI is the modality of choice for patients with a suspicious history, along with physical examination with examination of normal radiographs. The diagnosis of osteonecrosis on MRI was based on band‐like abnormal signals, and band‐like hypo‐intense zones on T1‐weighted images (Fig. 8). It is 99% sensitive and specific for detecting early shoulder osteonecrosis, which usually presents as an area of low‐intensity signal on T1‐weighted and high‐intensity signal onT2‐weighted images (Fig. 9). Bone marrow edema and a joint effusion may also be present. The later stages of shoulder osteonecrosis are better assessed using plain radiography and, in some cases, CT scans. CT scans are useful for evaluating patients with a suspected subchondral fracture, which may not be seen on MRI.

The percentage of the humeral head affected by the lesion can be calculated as the ratio of the volume of the lesion in relation to the volume of the humeral head, considered as half of a sphere. The extent of involvement may be graded as A, indicating mild (<15%), B, indicating moderate, and C, indicating severe extent (>30%), according to the percentage extent of the lesion in the humeral head.

**Fig. 10:**
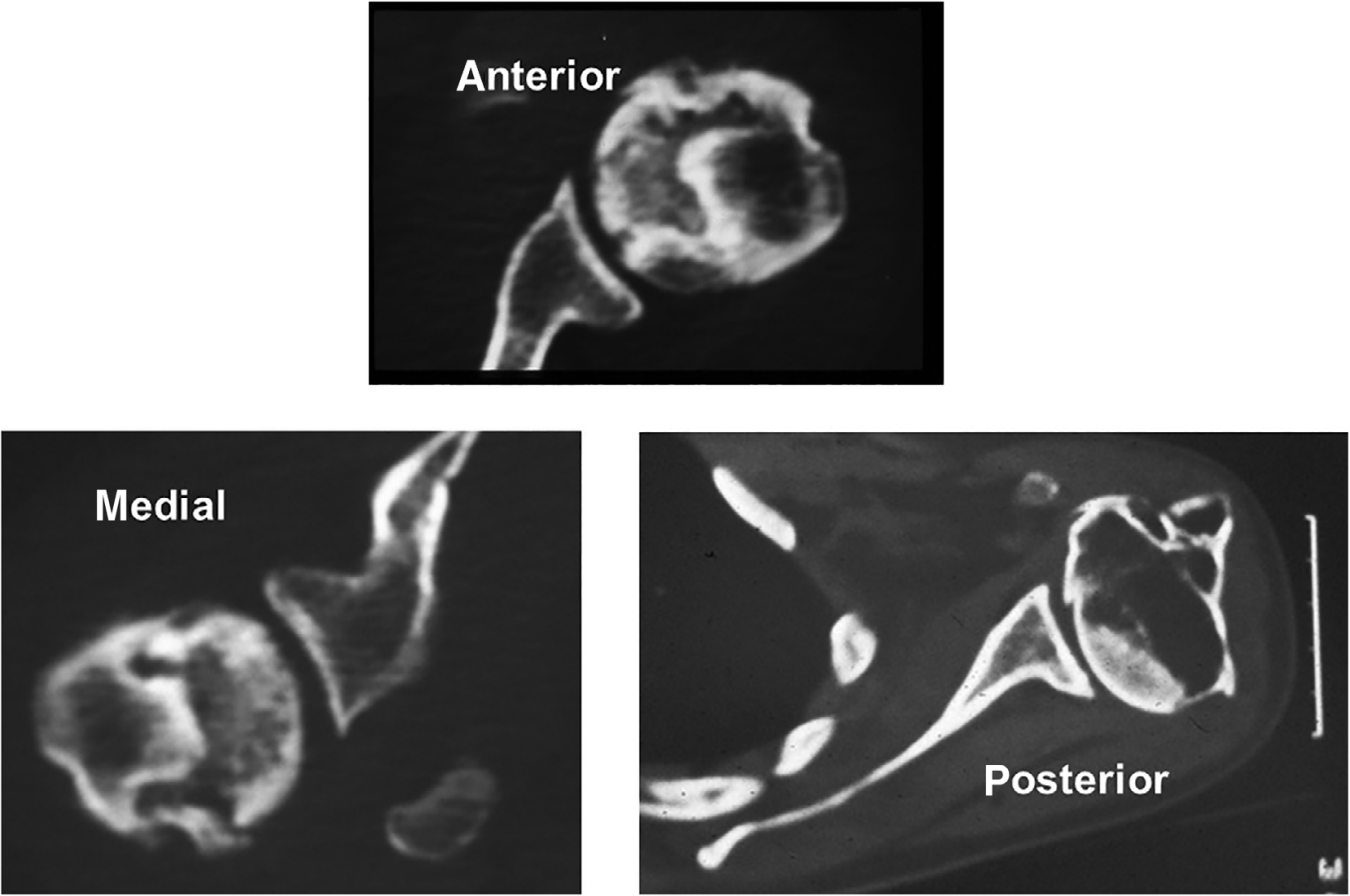
Different location of osteonecrosis on the humeral head.

The extent of the lesion in contact with the glenoid can be measured on the transverse image with MRI. The diameter of the glenoid may be divided into two parts: anterior and posterior. The necrotic area is expressed as anterior if in contact with less than half of the glenoid rim, posterior if in contact with less than half of the glenoid rim, and medial if in contact with all the glenoid rim (Fig. 10).

Once a lesion is identified, radiographs of the contralateral shoulder should be obtained. If the radiograph is negative, MRI should be considered. Alternatively, radionuclide imaging can be performed, to exclude disease in other joints.

## Classification

**Fig. 11:**
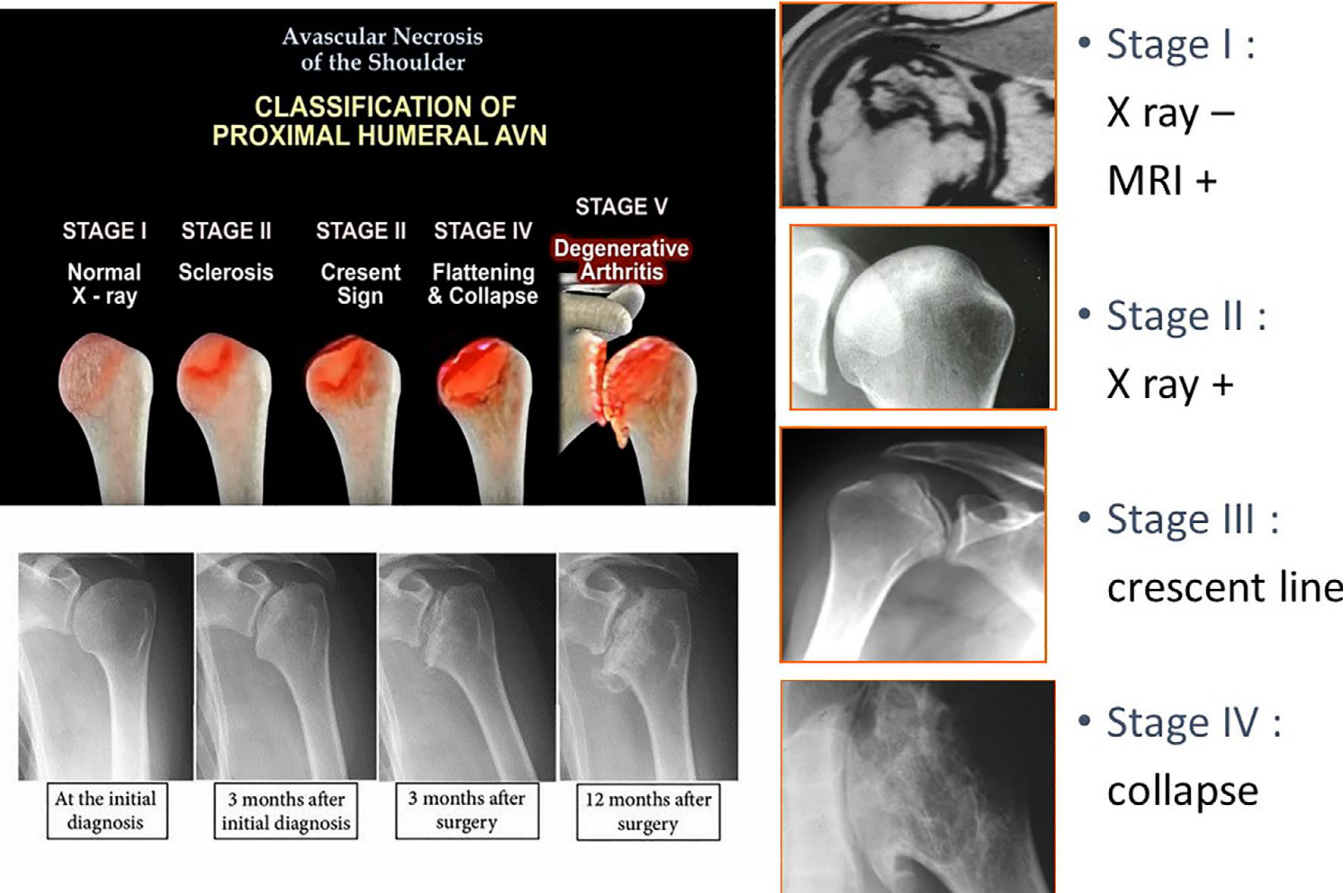
Classification of stages for shoulder osteonecrosis.

**Fig. 12:**
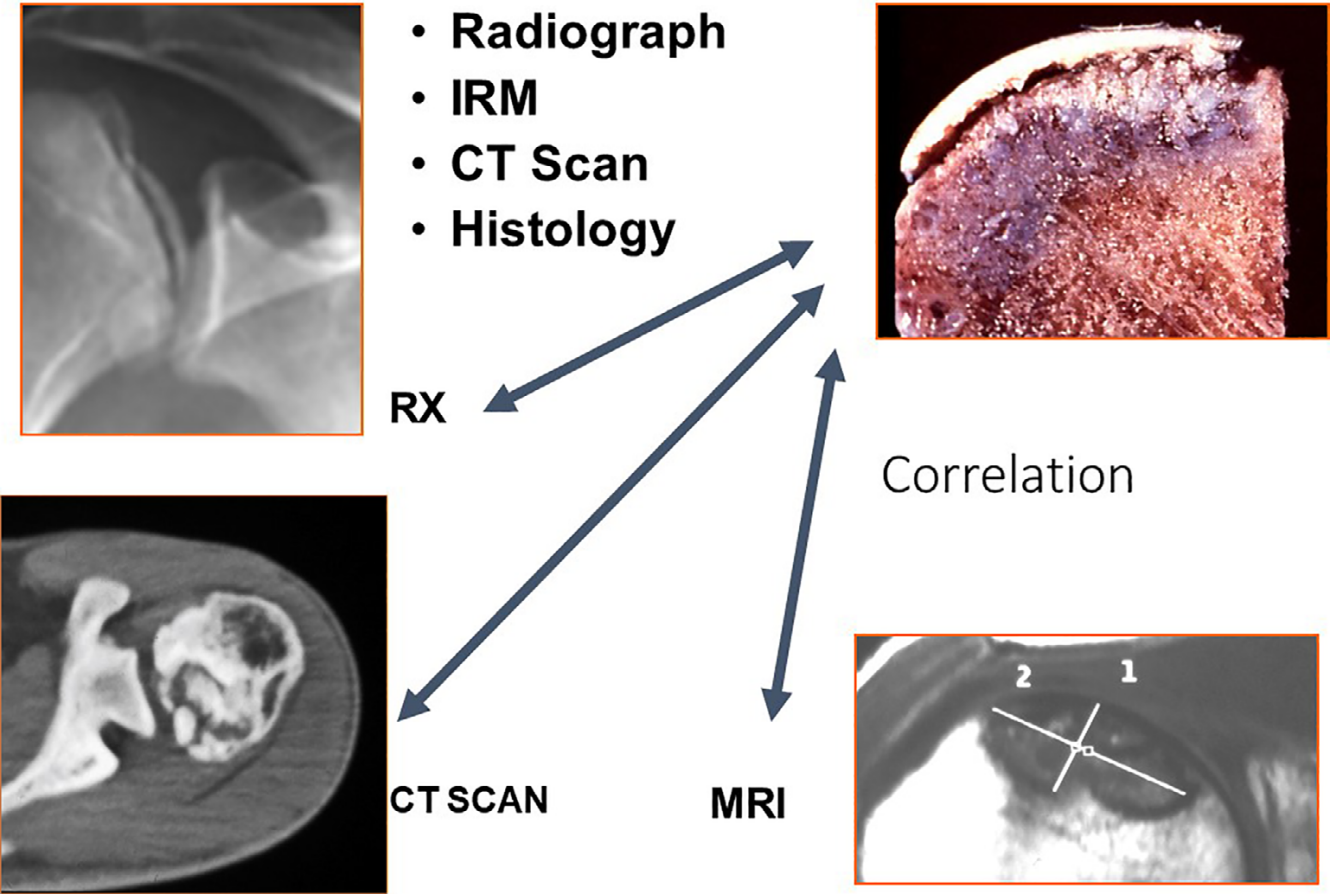
There is good correlation between different exams for stages.

Osteonecrosis of the humeral head is frequently classified using the description of Cruess (1978)^36^; it a variation of the system used by Ficat and Arlet^37^to describe hip osteonecrosis. It is split into five stages. Stage 1 is characterized by normal radiographs and abnormal MRI. The hyper‐intense areas of bone marrow are replaced by hypo‐intensity on T1‐weighted images, while a hyper‐intense focus is seen in T2 weighted images (Fig. 11). In stage 2, a reparative process, including sclerotic or diffusely mottled osteopenia, is seen; sphericity of the humeral head is maintained. Stage 3 is characterized by the crescent sign. It is a subchondral radiolucent line; in this stage, the subchondral collapse may also cause minor depressions in the joint surface. Stage 4 disease has collapse where the articular surface presents destruction of the underlying trabecular pattern. Osteo‐cartilagenous flaps of bone may occur and break free to become loose intra‐articular bodies. Stage 5 is when the glenoid has developed articular changes due to joint incongruity. Other authors have proposed similar classifications^4^, ^38^.There is a correlation between different exams and histology (Fig. 12).

## Treatment

Conservative treatment options are proposed and effective in patients with stage I and II osteonecrosis. Two types of core decompression have been described: a percutaneous technique using a classical delto‐pectoral approach or an arthroscopic technique. After collapse, the indication is usually an arthroplasty. Osteonecrosis as a surgical indication accounts for approximately 5% of all shoulder arthroplasties performed. However, relatively few studies have evaluated the outcomes following arthroplasty for osteonecrosis of the humeral head and many studies include only a few cases or have a short‐term follow‐up. The decision to use hemi‐arthroplasty or a total shoulder arthroplasty is usually based on the glenoid status and the surgeon's opinion. One absolute contraindication for arthroplasty is active infection, and relative contraindications include significant brachial plexus injury and concomitant deltoid and rotator cuff insufficiency, where a reverse shoulder arthroplasty can be proposed. The challenge when it comes to shoulder arthroplasty in patients with atraumatic osteonecrosis remains prosthesis longevity, as these patients are relatively young.

### *Conclusion*

shoulder osteonecrosis is a complex condition that is not fully understood. It most commonly arises from trauma or corticosteroid and alcohol use, but is also associated with a variety of other risk factors, including blood dyscrasias and metabolic and coagulation disorders. Initial evaluation of the shoulder should include a thorough history and physical examination, as well as assessment of plain radiographs of the hip and pelvis. Early stage shoulder osteonecrosis is best evaluated by MRI, and CT scans can be helpful in identifying subchondral fractures.
